# The Coronavirus pandemic and closed fitness clubs negatively affected members exercise habits

**DOI:** 10.3389/fspor.2022.985782

**Published:** 2022-11-23

**Authors:** Christina Gjestvang, Elene Mauseth Tangen, Lene A. H. Haakstad

**Affiliations:** Department of Sports Medicine, Norwegian School of Sports Sciences, Oslo, Norway

**Keywords:** Body Mass Index, closed gyms, COVID-19, COVID-19 lockdown, exercise attendance, exercise behavior

## Abstract

**Introduction:**

Due to the Coronavirus pandemic, politicians enacted directions to reduce social interactions, including lockdown of fitness clubs. We aimed to investigate how this changed exercise habits of Norwegian gym members.

**Method:**

Based on survey data, men and women (≥18 years, *n* = 233, data collection from Aug. 2020 to Jan. 2021) were recruited to this study by an email-invitation from their fitness club chain or by Facebook advertisement. The participants reported on background variables (e.g., age, gender, total household income, occupation, and education), and exercise habits pre- and during social lockdown. Data were analyzed using independent or student *t*-test, chi-squared test, or McNemar's test, as appropriate.

**Results:**

Home-based exercise (18.0 vs. 72.5%, *p* = <0.001), walking (49.8 vs. 65.2%, *p* = <0.001), and cycling (16.7 vs. 24.5%, *p* = 0.004) was more common during than pre-lockdown. Also, men (4.33 to 3.68 days/week, *p* =0.013) and women (4.20 to 3.79 days/week, *p* = 0.001) reported a lower exercise frequency, and a shorter duration. Exercise frequency was lower in those with a BMI ≥25 than in those with BMI <25 (3.95 vs. 4.48 days/week, *p* = <0.007) pre-lockdown. High exercise attendees (≥3 sessions/week, 66.5%) reported a smaller decrease in exercise frequency (mean change: 0.06 vs. 1.24 days/week, *p* = <0.001) and duration (>60 min. per session: 33.0 vs. 3.8%, *p* = <0.001) than low exercise attendees during lockdown.

**Discussion:**

Home-based exercise, walking, and cycling were most frequently reported during lockdown. Participants reported a small decrease in exercise duration and frequency compared with pre-lockdown. Closure of fitness clubs impacted low attendees more than high attendees.

## Introduction

In 2020, an outbreak of the Coronavirus disease (COVID-19) originated worldwide and caused a high number of individuals with COVID-19 and associated deaths (1). An uncontrolled spread of the virus would have substantially exceeded the capacity of the healthcare system (1), and there was a need to slow the pandemic down (2). Most European countries implemented rules and legislations to reduce social interactions, a key factor to slow down respective outbreaks (3, 4). On 12 March 2020, the Norwegian health authorities and politicians shut down businesses and public infrastructure, including sports and leisure facilities such as fitness clubs, sports clubs, swimming pools, and community sports grounds. While these measures were important to decrease the spread of COVID-19, several studies have shown that these restrictions negatively affected physical activity habits among the general adult population (5–9). For instance, studies have shown that COVID-19 lockdown resulted in a decrease in all intensity levels of physical activity (vigorous, moderate, walking, and overall), lower compliance with the physical activity recommendations (from 81 to 62%), as well as a parallel increase in daily sitting time of 28% (from 5 to 8 h per day) (9, 10).

Access to exercise arenas that are available and convenient, such as fitness clubs, is described as important to facilitate regular participation in physical activity (11, 12). Based on data from The Global Health and Fitness Association (IHRSA) (13), before the Coronavirus pandemic, the global fitness club industry had about 184 million members and counted more than 210 000 gyms. In Norway, about one million members belongs to nearly 1200 gyms (14, 15). A fitness club holds equipment for group and individual exercise and offers a wide range of exercise concepts designed to encourage individuals of all ages to participate in regular exercise. Even though evidence suggests that individuals in the general adult population became less active and increased sedentary time during the pandemic, it is unclear whether the government‘s restrictions influenced exercise behavior among those who are members of a fitness club, an important exercise facility for many individuals. Fitness club members may be more motivated to be physically active than other individuals, due to e.g., a financial commitment (monthly membership fee), different exercise concepts in a safe indoor environment, and access to exercise guidance (11, 16).

A literature search revealed only two (17, 18) published studies in this field. Alexandris et al. (18) examined fitness club members' (n 323) willingness to start exercising in the clubs after the COVID-19 lockdown and found that 33 % did not intend to return when the gyms were allowed to re-open. The qualitative study of Kaur et al. (17) conducted semi-structured telephone interviews among 22 regularly active fitness club members and found that the participants experienced a lack of exercise motivation during COVID-19 lockdown compared with pre-lockdown. In the study of Kaur et al. (17) one disadvantage was the qualitative approach with a lack of measures and statistical representation, only giving perspectives from 22 individuals (19). Further, Alexandris et al. (18) did not investigate exercise behavior pre- and during COVID-19 lockdown, or if the members recovered their exercise behavior. Based on available scientific literature, fitness club members‘ exercise behavior during the pandemic are an underexplored research area. These two studies also call for more research, regarding if fitness club members have changed their exercise behavior as a result of COVID-19, and if they have recovered after the end of the lockdown.

A fitness club has equipment not available to most in a home-based setting, and as such facilitates engagement in e.g., resistance exercise, a common workout mode at fitness clubs (20). Even though the closing of fitness clubs was inevitable to cope with the pandemic, it forced millions of members to stay at home and limited their opportunities to engage in exercise. The fitness club members thereby lost their opportunity to receive exercise supervision by fitness instructors. Also, exercise barriers, such as a lack of knowledge, infrastructure, and motivation may have increased even more during the shutdowns of fitness clubs (21). Thus, leading to decreased levels of exercise in this specific population (22). Further, the fitness club environment may foster a sense of motivation, a key factor influencing exercise behavior (23). For instance, before COVID-19, one study found that fitness club members were ten times more likely than non-members to meet the recommendations of muscle-strengthening activities in at least two sessions/week (24). The fitness club industry is in a unique position to implement motivational principles into practice, that can support members' exercise motivation. Self-determination theory (SDT) (25, 26) proposes that when individuals' basic psychological needs for autonomy, competence, and relatedness are met, self-determined motivation improves. In the fitness club context, several studies have reported that when focusing on autonomy (e.g., enjoyable exercise experiences), competence (e.g., attend at group exercise classes or use of a personal trainer), and relatedness (e.g., social interaction and having friends at the gym) exercise attendance increased among members (27–29). How fitness clubs satisfy the members' three basic needs to increase exercise motivation and help members break down common barriers is important for exercise behavior (27). Hence, we anticipated that closed gyms, limited access to exercise equipment, as well as lack of space and infrastructure for home-based exercise led to a negative influence on exercise behavior in this population.

Worldwide, overweight (Body Mass Index (BMI) ≥25) and obesity (BMI ≥ 30) are some of the largest public health challenges (32). It is well known that a high BMI and physical inactivity increase the risk of several non-communicable diseases and all-cause mortality, and is among the most important causes of the increase in overweight and obesity (33–35). Regular participation in physical activity is therefore especially important for overweight and obese individuals with an additional risk of diseases such as diabetes type 2 and metabolic syndrome (36). We have previously reported a high number of overweight individuals exercising in fitness clubs (37, 38) and many fitness club members also report weight loss as one reason for exercise (39–41). Fitness clubs typically have personnel who can assist members with exercise program design, nutrition information, and weight management advice (42). We anticipated that individuals with a BMI ≥25 may be to a greater extent impacted by the lockdown of fitness club compared with those with a BMI <25. A recent study by Robinson et al. (6) reported that a high BMI negatively influenced and predicted lower physical activity levels during lockdown among overweight and obese UK adults. Yet, the impact of the lockdown on exercise habits in overweight and obese fitness club members remains unclear.

This study may provide a valuable snapshot into some ways in which the COVID-19 lockdown affected exercise behavior in a specific sample of fitness club members. The primary aim of the current study was to compare exercise habits (exercise frequency, duration, and type of activity) between men and women, and between those with a BMI <25 and BMI ≥25 in a group of fitness club members pre- and during lockdown in Norway, spring 2020. In addition, we wanted to compare exercise habits and motivation between the most active fitness club members (high attendees, ≥3 sessions/week) and those using the fitness club less frequently (low attendees, <3 sessions/week). We hypothesized that lockdown of fitness clubs negatively influenced members exercise habits.

## Materials and methods

This paper is a cross-sectional analysis of start-up data from a 1-year prospective study aiming to investigate differences in background and health variables, exercise behavior, motivation, barriers, self-efficacy, and social support between members from different fitness club segments. Cross-sectional analyses in this study were mainly based on data collected at start-up, and also at 3 months follow-up. The study was conducted in accordance with the Declaration of Helsinki and approved by the Norwegian Center for Research Data (NSD 296859). The research received no external funding.

Participants were recruited between August 2020 and November 2020 from three different fitness club segments: multipurpose (resistance and cardio exercise rooms, group exercise classes, and a wide range of exercise concepts, middle to high membership fee), fitness-only (resistance and cardio exercise rooms for individual exercise, low membership fee), or boutique fitness clubs (one or two specialized exercise concepts, high membership fee). All were invited to take part in the study by an email-invitation from their fitness club chain or by an advertisement at Facebook. By a link in the email-invitation or Facebook advertisement, all participants gave informed consent to participate in the web-based system SurveyXact 8.2 (Ramböll, Aarhus, Denmark). The electronic informed consent contained study information according to the Helsinki Declaration and data was non-identifiable. The IT department at the Norwegian School of Sport Sciences provides storage services, and Norwegian regulations require that all raw research data should be kept for at least 5 years after study completion.

In an attempt to keep a count on the response rate, we first distributed recruitment directly per email to chief executive officers (CEOs) in chosen fitness club chains. The CEOs were asked to respond with the number of members who received information regarding our study and link for participation. Unfortunately, most CEOs did not provide the actual number of invited members, and the number of recruited participants was also not satisfactory. Thus, we decided to recruit more widely on social media. Eligibility criteria were: ≥18 years, member at one of the three fitness club segments, and motivated to respond to an electronic questionnaire at four time-points across 12 months (at baseline, and after three, six, and 12 months). In total 233 members responded to recruitment and responded to the questionnaire at baseline.

A section from the questionnaire (a total of 64 questions) used in the main study was used to answer the present study aims. All questions were close-ended, and the survey as a whole took approximately 25 min to complete. For the present study aims, background variables, exercise motivation, and six questions developed by the project group concerning physical activity and exercise habits were asked at start-up retrospectively based on an average week pre- and during social lockdown (Table 1). To investigate whether the participants recovered their exercise frequency, we also used self-reported exercise frequency obtained at 3 months follow-up (*n* = 204) in the main study.

**Table 1:** Questions used to answer the present study aims.

**Specifics**	**Statements/** **Questions**	**Response options**
To obtain data on exercise motivation a Norwegian version of the validated Behavioral Regulation in Exercise Questionnaire-2 (BREQ-2) was used (30), where the individuals rate the significance of 19 statements as a personal motive to engage, or not engage in exercise. Statements were divided into five subscales, and a sum score (from 0 to 4) for each subscale was calculated by adding scores from each statement, divided by the number of statements	**Intrinsic regulation:** “I exercise because it's fun,” “I enjoy my exercise sessions,” “I find exercise a pleasurable activity,” “I get pleasure and satisfaction from participating in exercise,” **identified regulation:** “I value the benefits of exercise,” “It's important to me to exercise regularly,” “I think it is important to make the effort to exercise regularly,” “I get restless if I don't exercise regularly,” **introjected regulation:** “I feel guilty when I don't exercise,” “I feel ashamed when I miss an exercise session,” “I feel like a failure when I haven't exercised in a while,” **external regulation:** “I exercise because other people say I should,” “I take part in exercise because my friends/family/partner say I should,” “I exercise because others will not be pleased with me if I don't,” “I feel under pressure from my friends/family to exercise,” **amotivation:** “I don't see why I should have to exercise,” “I can't see why I should bother exercising,” “I don't see the point in exercising,” “I think exercising is a waste of time”	“*0 not true for me,” “1,” “2 partly true for me,” “3,” or “4 very true for me”*
To determine whether participants were meeting the physical activity recommendations, a	Physical activity is recommended for a minimum of 2.5 h per week of moderate intensity. E g., 30 min	“Yes” or “No”
question based on intensities described in the International Physical Activity Questionnaire (IPAQ) (31) was asked	of moderate intensity 5 times a week and includes all activity (including exercise) that makes you sweat and breathe somewhat harder than normal. Based on this, would you characterize yourself as physically active?	
To determine whether participants had been exercising	Exercise is physical activity that is planned, structured, and repetitive for the purpose of conditioning the body. Based on this, have you exercised 1 session/week (≥60 min.) or more the last 6 months?	“Yes” or “No”
To determine participants exercise frequency	Before/during COVID-19 social lockdown, how often did you exercise?	“0 days/week,” “1 day/week,” “2 days/week,” “3 days/week,” “4 days/week,” “5 days/week,” “6 days/week,” or “Every day”
To determine participants exercise duration	Before/during COVID-19 social lockdown, for how long did you usually exercise per session?	“ < 30 min,” “31 to 45 min,” “46 to 60 min,” “61 to 75 min,” “76 to 90 min,” or “>90 min” In the analyses, the response options were merged into following categories: < 60 min, 61 to 89 min, and >90 min
To determine participants type of activity	Before COVID-19 social lockdown, what kind of activity did you usually do? Participants were able to select more than one response.	“Exercise at the fitness club,” “exercise at a sports club (such as handball, soccer, cross-country skiing og swimming), “commuting activities (such as walking or cycling to and from work/school),” “activities at home
		(such as housework or gardening),” “walking,” “cycling,” “exercise at home,” or “other activities”
	During COVID-19 social lockdown, what kind of activity did you usually do? Participants were able to select more than one response.	“Exercised at calisthenics parks,” “commuting activities (such as walking or cycling to and from work/school),” “activities at home (such as housework or gardening),” “walking,” “cycling,” “exercise at home,” or “other activities”

Data were analyzed using SPSS (IBM Corp. Released 2016. IBM SPSS Statistics for Windows, Version 24.0. Armonk, NY: IBM Corp). An independent or student *t*-test for continuous variables and chi-squared test or McNemar's test for proportions were used as appropriate. To address if changes in exercise habits was different in participants having a BMI ≥25 compared with those having a BMI <25, we divided the participants into BMI groups [BMI <25 and BMI ≥25, based on self-reported body weight and height (questionnaire data)]. For the same purpose, we also divided the participants into high (≥3 exercise sessions/week) and low (<3 exercise session/week) exercise attendance (43). We also calculated Hedges g effect size, with values around 0.2, 0.5 and 0.8 interpreted as weak, medium and strong effect sizes, respectively. Results are presented as means ± SD, or frequencies (n) and percentages, mean change, and 95% CI for mean change. A two-tailed alpha level of 0.05 was used for statistical significance.

## Results

General characteristics of participants are shown in Table 2. Almost half (45.1%) were ≤35 years, whereas 5.6% were aged ≥65. Membership length were reported as follows: <4 weeks: 4.3%, 2 to 12 months: 18.9%, 1 to 5 years: 41.2%, more than 5 years: 18.0%, and more than 10 years: 17.6%. A larger proportion of men had a BMI ≥25 compared with women (52.5 men vs. 29.0% women, *p* = 0.007). Otherwise, men and women, and participants with a BMI <25 and BMI ≥25 were balanced in background variables. Pre-lockdown, most members reported to meet the physical activity recommendations of ≥2.5 h of moderate intensity per week (88.4%) the last 6 months pre-lockdown.

**Table 2:** General characteristics of participants.

	**All**	**Men**	**Women**		**BMI < 25**	**BMI ≥25**	
	**(*n* = 233)**	**(*n* = 40)**	**(*n* = 193)**		**(*n* = 134)**	**(*n* = 99)**	
**Variable**	**Mean (SD)**	**Mean (SD)**	**Mean (SD)**	***p***	**Mean (SD)**	**Mean (SD)**	***p***
Age (years)	39.62 (13.67)	41.85 (14.68)	39.16 (13.44)	0.257	39.26 (13.55)	40.10 (13.88)	0.644
Body weight (kg)	72.82 (13.47)	84.38 (11.93)	70.42 (12.51)	< 0.001	65.66 (8.52)	82.51 (12.88)	< 0.001
Body height (cm)	170.45 (7.99)	179.88 (9.53)	168.49 (6.02)	< 0.001	170.29 (8.03)	170.66 (7.96)	0.365
Mean BMI (kg/m^2^)	24.98 (3.80)	26.04 (2.68)	24.76 (3.97)	0.015	22.56 (1.55)	28.26 (3.48)	< 0.001
	***n (%)***	***n (%)***	***n (%)***		***n (%)***	***n (%)***	
High educational level (≥4 years)	94 (40.3)	19 (47.5)	75 (38.9)	0.403	57 (42.5)	37 (37.4)	0.510
High household income (>100 000$/year)	97 (41.6)	18 (45.0)	79 (40.1)	0.765	53 (39.6)	44 (44.4)	0.539
Spouse/partner	118 (50.6)	22 (55.0)	96 (49.7)	0.666	61 (45.5)	57 (57.6)	0.092
Having children	63 (27.0)	7 (17.5)	56 (29.0)	0.195	35 (26.1)	28 (28.3)	0.827
Full-time employment	138 (84.1)	29 (72.5)	109 (56.5)	0.187	80 (59.7)	58 (58.6)	0.626
**Membership length**				0.208			0.249
< 1 year	54 (23.2)	5 (12.5)	49 (25.4)		26 (19.4)	28 (28.3)	
1 to 5 years	96 (41.2)	18 (45.0)	77 (39.9)		59 (44.0)	36 (36.4)	
>5 years	83 (35.6)	17 (42.5)	67 (34.7)		49 (36.6)	35 (35.4)	

During lockdown, we observed that all participants worked out less frequently compared with pre-lockdown (mean change: 0.45 days/week, 95% CI 0.24, 0.67, *p* = <0.001, Table 3). The participants also reported shorter exercise duration during lockdown, compared with pre-lockdown (*p* = <0.001). For instance, 70.6% exercised > 60 min per session pre-lockdown, whereas this proportion decreased to 24.1% during lockdown (*p* = <0.001). The same habits were seen when analyzing men and women separately (Table 2). Domestic chores such as housework and gardening (activities at home 28.0 vs. 10.0%, *p* = 0.028) and walking (54.4 vs. 27.5%, *p* = 0.003) was more common in women than men pre-lockdown. During lockdown, walking (45.0 vs. 69.4%, *p* = 0.006) was more frequently reported in women.

**Table 3:** Comparison of exercise habits pre- and during lockdown.

	**Pre-social lockdown**	**During social lockdown**		**Pre-social lockdown**			**During social lockdown**	
	**All**	**All**	***p***	**Men**	**Women**	***p***	**Men**	**Women**	***p***
	**(*n* = 233)**	**(*n* = 233)**		**(*n* = 40)**	**(*n* = 193)**		**(*n* = 40)**	**(*n* = 193)**	
**Variables**	**Mean (SD)**	**Mean (SD)**		**Mean (SD)**	**Mean (SD)**		**Mean (SD)**	**Mean (SD)**	
Exercise frequency (days/week)	4.22 (1.50)	3.77 (1.91)	< 0.001	4.33 (1.47)	4.20 (1.50)	0.951	3.68 (1.90)	3.79 (1.92)	0.917
	***n*** **(%)**	***n*** **(%)**		***n*** **(%)**	***n*** **(%)**	***p***	***n*** **(%)**	***n*** **(%)**	***p***
**Exercise duration**									
< 60 min	124 (53.2)	169 (75.4)	< 0.001	17 (42.5)	107 (55.4)	0.187	28 (73.7)	141 (75.8)	0.944
61 to 89 min	83 (64.1)	44 (19.6)	< 0.001	17 (42.5)	66 (34.6)	0.441	7 (18.4)	37 (19.9)	1.000
>90 min	15 (6.5)	10 (4.5)	0.267	4 (10.0)	11 (5.8)	0.524	3 (7.9)	7 (3.8)	0.489
**Type of activity**									
Exercise at home	42 (18.0)	169 (72.5)	< 0.001	7 (17.5)	35 (18.1)	1.000	24 (60.0)	145 (75.1)	0.079
Walking	116 (49.8)	152 (65.2)	< 0.001	11 (27.5)	105 (54.4)	0.002	18 (45.0)	134 (69.4)	0.006
Cycling	39 (16.7)	57 (24.5)	0.004	7 (17.5)	32 (16.6)	1.000	12 (30.0)	45 (23.3)	0.488
Commuting activities	103 (44.2)	91 (39.1)	0.162	18 (45.0)	85 (44.0)	1.000	14 (35.0)	77 (39.9)	0.689
Activities at home	175 (75.1)	164 (70.4)	0.063	4 (10.0)	54 (28.0)	0.028	7 (17.5)	62 (32.1)	0.098
Other activities	35 (15.0)	40 (17.2)	0.472	8 (20.0)	27 (14.0)	0.468	8 (20.0)	32 (16.6)	0.771
Exercise at the fitness club	224 (96.1)	-	-	39 (97.5)	185 (97.9)	1.000	-	-	-
Exercise at a sports club	28 (12.0)	-	-	6 (15.4)	22 (11.8)	0.721	-	-	-
Exercise at calisthenics parks	-	111 (47.6)	-	-	-	-	20 (51.3)	91 (48.1)	0.857

Table 4 shows comparison of exercise habits pre- and during lockdown in BMI groups (BMI <25 and BMI≥25). Pre-lockdown, exercise frequency was lower in those with a BMI ≥25 than in those having a BMI <25 (3.95 vs. 4.48 days/week, 95% CI (mean change): −0.93, −0.14, *p* = <0.007). This difference was not found during lockdown, but more of those with a BMI ≥25 reported exercise dropout than those having a BMI <25 (7.1 vs. 1.5%, *p* = 0.066).

**Table 4:** Comparison of exercise habits pre-and during lockdown between BMI groups.

	**Pre-social lockdown**				**During social lockdown**		
	**BMI < 25**	**BMI ≥25**			**BMI < 25**	**BMI ≥25**		
	**(*n* = 134)**	**(*n* = 99)**			**(*n* = 134)**	**(*n* = 99)**		
**Variables**	**Mean (SD)**	**Mean (SD)**	***p***	**Hedges g**	**Mean (SD)**	**Mean (SD)**	***p***	**Hedges g**
Exercise frequency (days/week)	4.48 (1.32)	3.95 (1.66)	0.007	0.36	3.10 (1.06)	2.91 (1.08)	0.207	0.18
	***n*** **(%)**	**(%)**			***n*** **(%)**	***n*** **(%)**		
**Exercise duration**								
< 60 min	70 (52.2)	54 (54.5)	0.829		95 (72.0)	74 (80.4)	0.197	
61 to 89 min	53 (39.6)	30 (30.9)	0.226		31 (23.5)	13 (14.1)	0.118	
>90 min	9 (6.7)	6 (6.2)	1.000		6 (4.5)	4 (4.3)	1.000	
**Type of activity**								
Exercise at home	32 (23.9)	10 (10.1)	0.011		103 (76.9)	66 (66.7)	0.115	
Walking	74 (55.2)	42 (42.4)	0.072		89 (66.4)	63 (63.6)	0.763	
Cycling	29 (21.6)	10 (10.1)	0.031		38 (28.4)	19 (19.2)	0.146	
Commuting activities	60 (44.8)	43 (43.4)	0.944		55 (41.0)	36 (36.4)	0.556	
Activities at home	40 (29.9)	18 (18.2)	0.060		43 (32.0)	26 (26.3)	0.413	
Other activities	21 (15.7)	14 (14.1)	0.890		26 (19.4)	14 (14.1)	0.380	
Exercise at the fitness club	131 (97.8)	93 (93.4)	0.206		-	-	-	
Exercise at a sports club	18 (13.4)	10 (10.1)	0.639		-	-	-	
Exercise at calisthenics parks	-	-	-		76 (56.7)	35 (35.4)	0.004	

High exercise attendees (66.5%) had a mean exercise frequency of 4.81 (1.15) (pre-lockdown) and 4.75 (1.30) (during lockdown) days/week (*p* = 0.523). Low exercise attendees reported a decreased exercise frequency from pre- to during lockdown (1.81 (1.33) days/week, mean change: 1.24 days/week, *p* = <0.001, hedges g = 2.47). Also, low exercise attendees tended to work out shorter during lockdown than high exercise attendees (<60 min/session: 83.3 vs. 67.1%, *p* = <0.001). High attendees reported longer exercise durations than low attendees during lockdown (e.g., 61 to 89 min/session: 26.5 vs. 3.8%, *p* = <0.001). None of low attendees reported to work out >90 min (high attendees: 6.5%, *p* =0.031). Also, a higher proportion of high exercise attendees compared with low exercise attendees reported outdoor cycling and exercise at calisthenics parks during lockdown (outdoor cycling: 30.3 vs. 12.8%, *p* = 0.006 and exercise at calisthenics parks: 62.7 vs. 20.0%, *p* = <0.001). We found differences between high and low exercise attendees in the stages of the self-determination continuum with respect to exercise motivation (BREQ-2). High exercise attendees scored higher on intrinsic [3.56 (0.59) vs. 3.35 (0.70), *p* = 0.014] and identified [3.50 (0.52) vs. 3.37 (0.62), *p* = 0.047] regulation than low exercise attendees. However, the effect size was small (Hedges g: 0.33). No differences were found in introjected or external regulation, or amotivation.

We also investigated whether the participants recovered their exercise frequency. Compared with pre-lockdown, participants at 3 months follow-up reported an exercise frequency of 3.85 (1.57) days/week (*p* = <0.001, hedges g = 0.87). High exercise attendees reported a higher exercise frequency compared with low exercise attendees at 3 months follow-up [4.43 (1.43) vs. 2.64 (1.10), *p* = <0.001].

## Discussion

In the present study, among fitness club members, both men and women reported a reduction in exercise duration and frequency during lockdown compared with pre-lockdown. Both sexes mainly exercised at home, while more women than men also reported walking as a mode of exercise during lockdown. We found that exercise frequency was lower in those with a BMI ≥25 compared with those having a BMI <25 pre-lockdown, but this was not observed during lockdown. More participants with a BMI ≥25 reported exercise dropout than participants with a BMI <25. Our results are in line with our expectations, that the shutdown of fitness clubs negatively influenced exercise frequency and duration among gym members. Yet, when comparing participants with high and low exercise attendance, we found that closed gyms only affected those with low exercise attendance. Further, high exercise attendees reported slightly higher intrinsic and identified motivational regulation than low exercise attendees. This may indicate that low-active individuals are more vulnerable to closure of physical activity arenas such as fitness clubs than high-active individuals.

Among all participants, exercise frequency decreased by 0.5 days/week from pre- to during lockdown. In accordance with Brand et al. (44), we observed that the participants exercised for a shorter time during lockdown than pre-lockdown. A total of 64.1% exercised >61 min pre-lockdown, while the majority (75.4%) exercised <60 min during lockdown. Our findings are in line with previous research proposing that closed fitness clubs were associated with negative changes in exercise habits among members (17). In contrast to this, adults have reported more time to exercise during lockdown (45), and time constraints have previously been reported as a main barrier for regular exercise among fitness club members (38). Even though adults possibly had more leisure time for exercise during lockdown (45), closure of fitness clubs eliminates a comfortably climate-controlled exercise arena that offers a variety of exercise equipment and concepts, and personal trainers and fitness instructors providing exercise supervision (11, 20). Even though home exercise, including online and multimedia training approaches, are proposed as feasible, several adults have reported a lack of interest, a reduced effort during exercise, and a perception of less effective exercise during lockdown (17, 18, 46). Regrettably, we did not collect data regarding the type of home exercise or how the participants perceived this kind of activity.

Kaur et al. (17) found that fitness club members altered their exercise habits due to closed fitness clubs during lockdown. Thus, temporarily closed arenas for physical activity may force exercise behavioral change (47). In agreement with our findings, substituting exercise at different activity arenas with home-based exercise and outdoor activities was the main change in exercise behavior among the general population in Greece, United Kingdom, Ireland, New Zealand, and Australia during lockdown (18, 47). Similar results were found in a Norwegian study, where lockdown was associated with more recreational activities, with walking and use of outdoor green spaces as preferred activities (48).

Considering exercise duration and frequency, we did not observe any differences between men and women, in discrepancy with several studies that have observed more positive changes in exercise behavior during lockdown among women (47, 49–51). It is also previously reported that men are generally more active than women during normal circumstances, yet, it is reported that men had a higher drop in physical activity participation than women during lockdown (47, 49–51). An explanation for dissimilar findings compared with other research may be the unbalanced sex distribution in the present study (men *n* = 40, women *n* = 193). It may be that we had limited statistical power for a meaningful comparison between sexes. The unbalanced sex distribution may have increased the risk of type 1 error and reduced the chance to reveal small differences between men and women. Regarding type of activity, during lockdown, more women (69%) than men (45%) were engaged in walking. In agreement, Lopez-Bueno et al. (52) also found women to engage in more low-intensity activity (walking) during the initial period of lockdown than men.

We did not find a difference in exercise frequency between those with a BMI <25 and BMI ≥25 during lockdown, but more participants in the high BMI range reported exercise dropout compared with normal weight. Flanagan et al. (53) also found no difference in physical activity level between BMI group ≥25 and <25 during lockdown. Inconsistent with our findings, BMI was previously found as a predictor for lower physical activity levels during lockdown, and adults with overweight or obesity had a higher risk of developing unhealthy weight-related behaviors (6). Another study found adults with a BMI <25 to increase their physical activity level during lockdown, while those with BMI ≥25 had no significant change (54).

The shutdown of fitness clubs was not associated with negative changes in exercise behavior among high exercise attendees, defined as exercise ≥3 sessions/week. This underlines that exercise probably was easier to maintain during lockdown for fitness club members who already had a habit of regular exercise pre-lockdown, especially since high exercise attendees were more autonomously motivated than low exercise attendees. High exercise attendees possibly exercised for enjoyment, and further experienced satisfaction from exercise, two key factors that positively influence regular participation in exercise (55). In line with our findings, previous research has shown greater odds for regular exercise during lockdown among adults with high exercise attendance pre-lockdown (44, 56). It is also reported that those who met the recommendations for physical activity pre-lockdown generally met the recommendations during lockdown as well (44, 56). Further, in a study of German adults, those that remained active during lockdown (33%) reported home-based exercise during lockdown (57), which is in accordance with our findings. More high attendees than low attendees in our study also reported exercise at calisthenics parks and cycling during lockdown. Differences in exercise habits among high and low attendees might be seen as a consequence of less knowledge about exercise and lack of equipment (such as a bike and barbells) among low attendees. Oppositely, two studies among the general adult population found that low active adults reported being more physically active during lockdown compared to pre-lockdown (44, 57). Nevertheless, we believe that low active individuals who depend on fitness clubs might have challenges finding alternatives to “hitting the gym,” since they are used to exercise equipment and instructors providing exercise supervision.

It is consistently reported that social lockdown was associated with increased sedentary behavior, fewer commuting activities, as well as other unhealthy behaviors such as unfavorable eating habits (e.g., eating out of control or snacks between meals), and increased use of smartphones (9, 46, 52, 56). We found that low attendees were less active when the fitness clubs were closed than high exercise attendees. It is previously shown that among adults reporting to be less active during lockdown, closed infrastructure (such as fitness clubs and sports clubs) and absence of friends to be physically active with was reported as main barriers for physical activity during lockdown (45). On the other side, research tend to overlook the role of habits in self-regulation (58). Habits form by repeating a behavior, and high exercise attendees already had regular exercise routines as a part of their lifestyles, bypassing the need for conscious willpower during life events such as the Coronavirus pandemic.

The findings in the current study may be influenced by a majority of highly active individuals. Most (88.4%) of participants enrolled in the present study were following current physical activity recommendations pre-lockdown. Members who participated in this study were also mostly young to middle-aged, an age group that is reported to more easily maintain exercise during lockdown compared with older individuals (57). Thus, this may also explain why fitness club members‘ exercise frequency only decreased by 11% during lockdown. Yet, opposite results are found in a Spanish adult population, with younger individuals reporting decreased physical activity participation during lockdown (51). Hence, the evidence in this specific research field is somewhat mixed, and none has previously reported exercise habits pre- and during lockdown in fitness club members.

To our knowledge, this is one of the first studies investigating changes in exercise habits (exercise frequency, duration, and mode) in a sample of fitness club members pre- and during social lockdown in spring 2020, and also if they recovered their exercise behavior. Electronic questionnaires are cost-efficient, gather responses quickly, and may also be assumed as an appropriate measurement method during the COVID-19 pandemic. Hence, this may be considered as a strength of our study. Other aspects were a complete dataset, with no missing values, and that our participants included both men and women, even though the sex distribution was somewhat imbalanced (more women than men). Considering that the fitness club industry was shut down at the time the participants were enrolled, there is a possibility that we mainly enrolled fitness club members that were motivated to sustain their exercise habits during lockdown, and this affected our response rate. Hence, the representativeness of the present study results may be questionable. Other limitations include the self-reporting method which can be affected by recall or social desirability bias and lack of potential depth (59). For instance, self-report of body weight and height may be biased by the individual's feelings at the time they fill out the questionnaire and participants often want to present the best versions, or at least a socially acceptable version of themselves (60). Yet, we have previously published a paper showing that both male and female fitness club members reported body weight and height reasonably accurately and that BMI based on self-report appears to be a valid measure (61). Further, we believe that using a questionnaire to gather data on BMI was an appropriate measurement method during the COVID-19 pandemic and social lockdown. Regarding self-reported exercise behavior, the six questions concerning physical activity and exercise habits were self-developed and not based on previously validated questionnaires. This study originates from a Ph.D. study including another sample of fitness club members (37, 38, 62). One of the main purposes of the 1 year study was to be able to compare participants from the Ph.D. study (new members) and participants from the current study (more experienced members). Thus, for comparison analysis, the project group decided to re-use questions from the original project. Also, we did not assess exercise at calisthenic parks before lockdown in the questionnaire. We believed that this type of activity was not relevant for fitness club members before the shutdown of fitness clubs. However, having this response option at both time points would be relevant for comparing the numbers doing this type of activity both before and during lockdown. Further, we did not ask the participants to report what kind of exercise they conducted at home. Home-based exercise can be a multitude of exercises and obtaining such information would strengthen our study results. Further, we decided to recruit more widely through an advertisement on Facebook. This could bias our results, e.g., it may be that Facebook users are older than on other social media. It is shown that recruited participants may differ in demographic characteristics (e.g., age) across Internet recruitment mechanisms (63, 64). However, using social media seems to be appropriate for increasing the efficiency of recruitment efforts. Social media platforms are used at a high rate for daily information exchange, and offer the potential to reach a large audience, at low cost (63). Finally, unmeasured confounding variables may influence the association between exercise behavior and the COVID-19 lockdown. Also, due to the cross-sectional analysis, we cannot establish the direction of causality in our conclusions.

## Conclusion

With the results obtained in our sample, it seems that during lockdown, shutdown of fitness clubs in Norway was associated with slightly negative changes in exercise frequency and duration among fitness club members. Home-based exercise, walking, and cycling were most frequently reported during lockdown. No differences in exercise habits were observed between sexes, or BMI groups ≥25 and <25 during lockdown, but exercise dropout was more frequently reported among those in the high BMI range than normal weight. When comparing low and high exercise attendees, closed gyms seemed to impact those who were less active pre-lockdown only.

## Data availability statement

The raw data supporting the conclusions of this article will be made available by the authors, without undue reservation.

## Ethics statement

The studies involving human participants were reviewed and approved by the study was conducted in accordance with the Declaration of Helsinki and approved by the Norwegian Center for Research Data (NSD 296859). The patients/participants provided their written informed consent to participate in this study.

## Author contributions

Conceptualization and supervision: LH. Methodology: LH and CG. Formal analysis, investigation, and project administration: CG. Writing—original draft preparation: CG and ET. Writing—review and editing: CG, ET, and LH. All authors have read and agreed to the published version of the manuscript.

## Conflict of interest

The authors declare that the research was conducted in the absence of any commercial or financial relationships that could be construed as a potential conflict of interest.

## Publisher's note

All claims expressed in this article are solely those of the authors and do not necessarily represent those of their affiliated organizations, or those of the publisher, the editors and the reviewers. Any product that may be evaluated in this article, or claim that may be made by its manufacturer, is not guaranteed or endorsed by the publisher.

## References

[1] World Health Organization. Coronavirus Disease (COVID-19) Pandemic. (2020). Available online at: https://wwweurowhoint/en/about-us/regional-director/statements-and-speeches/2020/statement-every-country-needs-to-take-boldest-actions-to-stop-covid-19 (acessed15, March 2021).

[2] World Health Organization. Every Country Needs to Take Boldest Actions to Stop COVID-19. (2020). Available Online at: https://wwweurowhoint/en/about-us/regional-director/statements-and-speeches/2020/statement-every-country-needs-to-take-boldest-actions-to-stop-covid-19 (acessed15, March 2021).

[3] Ferguson NM Cummings DAT Fraser C Cajka JC Cooley PC Burke DS. Strategies for mitigating an influenza pandemic. Nature. (2006) 442:448–52. 10.1038/nature04795 16642006 PMC7095311

[4] Glass RJ Glass LM Beyeler WE Min HJ. Targeted social distancing design for pandemic influenza. Emerg Infect Dis. (2006) 12:1671–81. 10.3201/eid1211.060255 17283616 PMC3372334

[5] Lopez-Bueno R Calatayud J Andersen LL Balsalobre-Fernandez C Casana J Casajus JA. Immediate impact of the COVID-19 confinement on physical activity levels in Spanish adults. Sustainability-Basel. (2020) 12:145708. 10.3390/su1214570833343415

[6] Robinson E Boyland E Chisholm A Harrold J Maloney NG Marty L. Obesity, eating behavior and physical activity during COVID-19 lockdown: a study of UK adults. Appetite. (2021) 156:104853 10.1016/j.appet.2020.104853 33038479 PMC7540284

[7] Helsingen LM Refsum E Gjostein DK Loberg M Bretthauer M Kalager M. The COVID-19 pandemic in Norway and Sweden - threats, trust, and impact on daily life: a comparative survey. BMC Public Health. (2020) 20:3. 10.1186/s12889-020-09615-3 33097011 PMC7582026

[8] Dor-Haim H Katzburg S Revach P Levine H Barak S. The impact of COVID-19 lockdown on physical activity and weight gain among active adult population in Israel: a cross-sectional study. BMC Public Health. (2021) 21:11523. 10.1186/s12889-021-11523-z 34362319 PMC8343341

[9] Ammar A Brach M Trabelsi K Chtourou H Boukhris O Masmoudi L. Effects of COVID-19 home confinement on eating behaviour and physical activity: results of the ECLB-COVID19 International Online Survey. Nutrients. (2020) 12:512852. 10.1159/000512852 32481594 PMC7352706

[10] Wilke J Mohr L Tenforde AS Edouard P Fossati C Gonzalez-Gross M. A pandemic within the pandemic? physical activity levels substantially decreased in countries affected by COVID-19. Int J Env Res Pub Health. (2021) 18:2235. 10.3390/ijerph18052235 33668262 PMC7967678

[11] Riseth L Nost TH Nilsen TIL Steinsbekk A. Long-term members' use of fitness centers: a qualitative study. BMC Sports Sci Med R. (2019) 11:114. 10.1186/s13102-019-0114-z 30828457 PMC6383217

[12] Wendel-Vos W Droomers M Kremers S Brug J van Lenthe F. Potential environmental determinants of physical activity in adults: a systematic review. Obes Rev. (2007) 8:425–40. 10.1111/j.1467-789X.2007.00370.x 17716300

[13] International Health Racquet & Sportsclub Association (IHRSA) (2020). The IHRSA Global Report 2020. Available online at: https://www.ihrsa.org/publications/the-2020-ihrsa-global-report/# (07 05 21).

[14] Virke Aktiv helse. Treningssenterbransjen 2019. (2019). Available online at: https://www.virke.no/analyse/statistikk-rapporter/treningssenterbransjen/

[15] Breivik GRK. Fysisk aktivitet omfang, tilrettelegging og sosial ulikhet–en oppdatering og revisjon Oslo: Norges idrettshøgskole. (2017). Available online at: https://www.godeidrettsanlegg.no/publikasjon/fysisk-aktivitet-omfang-tilrettelegging-og-sosial-ulikhet

[16] Nikolajsen H Sandal LF Juhl CB Troelsen J Juul-Kristensen B. Barriers to, and facilitators of, exercising in fitness centres among adults with and without physical disabilities: a scoping review. Int J Environ Res Public Health. (2021) 18:147341. 10.3390/ijerph18147341 34299792 PMC8304633

[17] Kaur H Singh T Arya YK Mittal S. Physical fitness and exercise during the COVID-19 pandemic: a qualitative enquiry. Front Psychol. (2020) 11:590172. 10.3389/fpsyg.2020.590172 33250827 PMC7673425

[18] Alexandris K Karagiorgos T Ntovoli A Zourladani S. Using the theories of planned behaviour and leisure constraints to study fitness club members' behaviour after COVID-19 lockdown. Leisure Stud. (2021). 10.1080/02614367.2021.1975802

[19] Moser A Korstjens I. Series: Practical guidance to qualitative research. Part 1: Introduction. Eur J Gen Pract. (2017) 23:271–3. 10.1080/13814788.2017.1375093 29185831 PMC8816396

[20] Gjestvang C Stensrud T Paulsen G Haakstad LAH. Stay true to your workout: Does repeated physical testing boost exercise attendance? A one-year follow-up study accepted for publication. J Sports Sci Med. (2020) 20:35–44. 10.52082/jssm.2021.35 33707984 PMC7919353

[21] Burgess E Hassmen P Pumpa KL. Determinants of adherence to lifestyle intervention in adults with obesity: a systematic review. Clin Obes. (2017) 7:123–35. 10.1111/cob.12183 28296261

[22] Stankowski CL Trauntvein NE. Hall SL. I use the student recreation center, but i would use it more if: understanding male and female constraints to student recreation. Center Use Recreat Sports J. (2017) 41:55–66. 10.1123/rsj.2015-0026

[23] Ryan RM Deci EL. Intrinsic and extrinsic motivations: classic definitions and new directions. Contemp Educ Psychol. (2000) 25:54–67. 10.1006/ceps.1999.1020 10620381

[24] Schroeder EC Welk GJ Franke WD Lee DC. Associations of health club membership with physical activity and cardiovascular health. PLoS ONE. (2017) 12:1–13. 10.1371/journal.pone.0170471 28107459 PMC5249148

[25] Teixeira P Carraca E Markland D Silva M Ryan R. Exercise, physical activity, and self-determination theory: a systematic review. Int J Behav Nutr Phys Act. (2012) 9:78. 10.1186/1479-5868-9-78 22726453 PMC3441783

[26] Deci EL Ryan RM. Motivation, personality, and development within embedded social contexts: An overview of self-determination theory. In:Ryan RM., editors, The Oxford Handbook of Human Motivation. New York: Oxford University Press. (2012) p. 85-107.

[27] Vlachopoulos S Neikou E. A prospective study of the relationships of autonomy, competence, and relatedness with exercise attendance, adherence, and dropout. J Sports Med Phys Fitness. (2007) 47:475–82. 18091690

[28] Springer JB Lamborn SD Pollard DM. Maintaining physical activity over time: the importance of basic psychological need satisfaction in developing the physically active self. Am J Health Promot. (2013) 27:284–93. 10.4278/ajhp.110211-QUAL-62 23402228

[29] Cetinkalp ZK Lochbaum M. Flourishing, affect, and relative autonomy in adult exercisers: a within-person basic psychological need fulfillment perspective. Sports. (2018) 6:48. 10.3390/sports6020048 29910352 PMC6027547

[30] Markland D Tobin V A. modification to the behavioural regulation in exercise questionnaire to include an assessment of amotivation. J Sport Exercise Psy. (2004) 26:191–6. 10.1123/jsep.26.2.191

[31] Craig CL Marshall AL Sjostrom M Bauman AE Booth ML Ainsworth BE. International physical activity questionnaire: 12-country reliability and validity. Med Sci Sport Exer. (2003) 35:1381–95. 10.1249/01.MSS.0000078924.61453.FB 12900694

[32] Ekelund U Ward HA Norat T Luan J May AM Weiderpass E. Physical activity and all-cause mortality across levels of overall and abdominal adiposity in European men and women: the European Prospective Investigation into Cancer and Nutrition Study (EPIC). Am J Clin Nutr. (2015) 101:613–21. 25733647 10.3945/ajcn.114.100065 PMC4340064

[33] Afshin A Forouzanfar MH Reitsma MB Sur P Estep K Lee A. Health effects of overweight and obesity in 195 countries over 25 years. N Engl J Med. (2017) 377:13–27. 10.1056/NEJMoa1614362 28604169 PMC5477817

[34] Aune D Sen A Prasad M Norat T Janszky I Tonstad S. BMI and all cause mortality: systematic review and non-linear dose-response meta-analysis of 230 cohort studies with 3.74 million deaths among 30.3 million participants. BMJ-Brit Med J. (2016) 353:2156. 10.1136/bmj.i2156 27146380 PMC4856854

[35] Di Angelantonio E Bhupathiraju SN Wormser D Gao P Kaptoge S de Gonzalez AB. Body-mass index and all-cause mortality: individual-participant-data meta-analysis of 239 prospective studies in four continents. Lancet. (2016) 388:776–86. 10.1016/S0140-6736(16)30175-1 27423262 PMC4995441

[36] Lippi G Henry BM Bovo C Sanchis-Gomar F. Health risks and potential remedies during prolonged lockdowns for coronavirus disease 2019 (COVID-19). Diagnosis. (2020) 7:85–90. 10.1515/dx-2020-0041 32267243

[37] Gjestvang C Abrahamsen F Stensrud T Haakstad LAH. What makes individuals stick to their exercise regime? a one-year follow-up study among novice exercisers in a fitness club setting. Front Psychol. (2021) 12:638928. 10.3389/fpsyg.2021.638928 34122230 PMC8194699

[38] Gjestvang C Abrahamsen F Stensrud T Haakstad LAH. Motives and barriers to initiation and sustained exercise adherence in a fitness club setting–a one-year follow-up study. Scand J Med Sci Sports. (2020) 30:1796–805. 10.1111/sms.13736 32488898 PMC7497044

[39] Gjestvang C Stensrud T Haakstad LAH. Are changes in physical fitness, body composition and weight associated with exercise attendance and dropout among fitness club members? Longitudinal prospective study. BMJ Open Sport Exerc Med. (2019) 9:1–9. 10.1136/bmjopen-2018-027987 30987992 PMC6500212

[40] Mullen S Whaley D. Age, gender, and fitness club membership: Factors related to initial involvement and sustained participation. J Sport Exerc Psychol. (2010) 8:24–35. 10.1080/1612197X.2010.9671931

[41] Ulseth A. New opportunities—complex motivations: gender differences in motivation for physical activity in the context of sports clubs and fitness centers. IJAASS. (2008) 20:44–66.

[42] Rustaden AM Haakstad LAH Paulsen G Bø K. Effects of BodyPump and resistance training with and without a personal trainer on muscle strength and body composition in overweight and obese women-A randomised controlled trial. Obes Res Clin Pract. (2017) 11:728–39. 10.1016/j.orcp.2017.03.003 28392264

[43] Hawley-Hague H Horne M Skelton DA Todd C. Review of how we should define (and measure) adherence in studies examining older adults' participation in exercise classes. BMJ Open Sport Exerc Med. (2016) 6:6. 10.1136/bmjopen-2016-011560 27338884 PMC4932302

[44] Brand R Timme S Nosrat S. When pandemic hits: exercise frequency and subjective well-being during COVID-19 pandemic. Front Psychol. (2020) 1:57067. 10.3389/fpsyg.2020.570567 33071902 PMC7541696

[45] Constandt B Thibaut E De Bosscher V Scheerder J Ricour M Willem A. Exercising in times of lockdown: an analysis of the impact of COVID-19 on levels and patterns of exercise among adults in belgium. Int J Env Res Pub Health. (2020) 17:4144. 10.3390/ijerph17114144 32532013 PMC7312512

[46] Sanudo B Fennell C Sanchez-Oliver AJ. Objectively-assessed physical activity, sedentary behavior, smartphone use, and sleep patterns pre- and during-COVID-19 quarantine in young adults from Spain. Sustainability-Basel. (2020) 12:5890. 10.3390/su12155890

[47] Faulkner J O'Brien WJ McGrane B Wadsworth D Batten J Askew CD. Physical activity, mental health and well-being of adults during initial COVID-19 containment strategies: a multi-country cross-sectional analysis. J Sci Med Sport. (2021) 24:320–6. 10.1016/j.jsams.2020.11.016 33341382 PMC7711171

[48] Venter ZS Barton DN Gundersen V Figari H Nowell M. Urban nature in a time of crisis: recreational use of green space increases during the COVID-19 outbreak in Oslo, Norway. Environ Res Lett. (2020) 15 abb396. 10.1088/1748-9326/abb396

[49] Giustino V Parroco AM Gennaro A Musumeci G Palma A Battaglia G. Physical activity levels and related energy expenditure during COVID-19 quarantine among the sicilian active population: a cross-sectional online survey study. Sustainability-Basel. (2020) 12:4356. 10.3390/su12114356

[50] Gjaka M Feka K Bianco A Tishukaj F Giustino V Parroco AM. The effect of COVID-19 lockdown measures on physical activity levels and sedentary behaviour in a relatively young population living in Kosovo. J Clin Med. (2021) 10:763. 10.3390/jcm10040763 33672837 PMC7918337

[51] Castaneda-Babarro A Arbillaga-Etxarri A Gutierrez-Santamaria B Coca A. Physical activity change during COVID-19 confinement. Int J Environ Res Public Health. (2020) 17:878. 10.3390/ijerph17186878 32967091 PMC7558959

[52] Lopez-Bueno R Calatayud J Casana J Casajus JA Smith L Tully MA. COVID-19 Confinement and health risk behaviors in Spain. Front Psychol. (2020) 11:1426. 10.3389/fpsyg.2020.01426 32581985 PMC7287152

[53] Flanagan EW Beyl RA Fearnbach SN Altazan AD Martin CK Redman LM. The impact of COVID-19 stay-at-home orders on health behaviors in adults. Obesity. (2021) 29:438–45. 10.1002/oby.23066 33043562 PMC7675243

[54] Romero-Blanco C Rodriguez-Almagro J Onieva-Zafra MD Parra-Fernandez ML Prado-Laguna MD Hernandez-Martinez A. Physical activity and sedentary lifestyle in university students: changes during confinement due to the COVID-19 Pandemic. Int J Env Res Pub Health. (2020) 17:85567. 10.3390/ijerph17186567 32916972 PMC7558021

[55] Rodrigues F Bento T Cid L Neiva HP Teixeira D Moutao J. Can interpersonal behavior influence the persistence and adherence to physical exercise practice in adults? a systematic review. Front Psychol. (2018) 9:2141. 10.3389/fpsyg.2018.02141 30459690 PMC6232376

[56] Lopez-Valenciano A Suarez-Iglesias D Sanchez-Lastra MA Ayan C. Impact of COVID-19 pandemic on university students' physical activity levels: an early systematic review. Front Psychol. (2021) 11:624567. 10.3389/fpsyg.2020.624567 33519653 PMC7845570

[57] Mutz M Gerke M. Sport and exercise in times of self-quarantine: how Germans changed their behaviour at the beginning of the COVID-19 pandemic. Int Rev Sociol Sport. (2021) 56:305–16. 10.1177/1012690220934335

[58] Fiorella L. The science of habit and its implications for student learning and well-being. Educ Psychol Rev. (2020) 32:603–25. 10.1007/s10648-020-09525-1

[59] Steene-Johannessen J Anderssen SA Van der Ploeg HP Hendriksen IJM Donnelly AE Brage S. Are self-report measures able to define individuals as physically active or inactive? Med Sci Sport Exer. (2016) 48:235–44. 10.1249/MSS.0000000000000760 26322556 PMC6235100

[60] Althubaiti A. Information bias in health research: definition, pitfalls, and adjustment methods. J Multidiscip Health. (2016) 9:211–7. 10.2147/JMDH.S104807 27217764 PMC4862344

[61] Haakstad LAH Stensrud T Gjestvang C. Does *self-perception equal the truth when judging own body weight and height?* Int J Environ Res Public Health. (2021) 18:502. 10.3390/ijerph18168502 34444251 PMC8394179

[62] Gjestvang C Stensrud T Hansen BH Kolle E Haakstad LAH. Are fitness club members likely to meet the current physical activity recommendations? TSM. (2019) 3:75–83. 10.1002/tsm2.120

[63] Arigo D Pagoto S Carter-Harris L Lillie SE Nebeker C. Using social media for health research: Methodological and ethical considerations for recruitment and intervention delivery. Digit Health. (2018) 4:1–15. 10.1177/2055207618771757 29942634 PMC6016568

[64] Ramo DE Hall SM Prochaska JJ. Reaching young adult smokers through the Internet: Comparison of three recruitment mechanisms. Nicotine Tob Res. (2010) 12:768–75. 10.1093/ntr/ntq086 20530194 PMC2893296

